# Obesity in children and adolescents and the risk of ovarian cancer: A systematic review and dose‒response meta-analysis

**DOI:** 10.1371/journal.pone.0278050

**Published:** 2022-12-07

**Authors:** Nan Ding, Junyi Zhan, Youjin Shi, Tianci Qiao, Panpan Li, Tingting Zhang

**Affiliations:** 1 Shanghai University of Traditional Chinese Medicine, Shanghai, China; 2 Department of Gynecology, Yueyang Hospital of Integrated Traditional Chinese and Western Medicine, Shanghai University of Traditional Chinese Medicine, Shanghai, China; 3 Department of Hepatology, Shuguang Hospital Affiliated to Shanghai University of Traditional Chinese Medicine, Shanghai, China; 4 Department of Neurology, Yueyang Hospital of Integrated Traditional Chinese and Western Medicine, Shanghai University of Traditional Chinese Medicine, Shanghai, China; Florida International University, UNITED STATES

## Abstract

**Objective:**

The relationship between obesity in children and adolescents and the risk of ovarian cancer remains controversial. The aim of this meta-analysis was to explore the exact shape of this relationship.

**Methods:**

We conducted dose‒response meta-analyses of cohort and case‒control studies, including published studies derived from searches in the PubMed, Embase, Web of Science and Cochrane Library databases until October 2022. Pooled effect size estimates are expressed as relative risks (RRs) or odds ratios (ORs) with 95% confidence intervals (CIs) and were evaluated by fixed-effect models. A nonlinear dose‒response meta-analysis was performed by using a restricted cubic spline model.

**Results:**

After screening 4215 publications, 10 studies were included in the present meta-analysis. Overall analyses revealed statistically significant associations of obesity in children and adolescents with ovarian cancer (adjusted RR = 1.19, 95% CI: 1.11 to 1.28, P < 0.001). Moreover, the association was consistently significant in most subgroup analyses, for example, using geographic stratification, the results remained stable both in the Americas(RR = 1.11; 95% CI: 1.01 to 1.21; P = 0.022) and Europe (RR = 1.46; 95% CI: 1.21 to 1.77; P<0.001). For the dose‒response analyses, the risk of ovarian cancer increased with the degree of obesity, and the trend increased rapidly when body mass index (BMI) was over 25.95 kg/m^2^.

**Conclusion:**

Our findings indicate that obesity in children and adolescents is a risk factor for ovarian cancer, and the risk increases with increasing BMI.

## Introduction

Ovarian cancer is the seventh most common cancer and the eighth leading cause of cancer death in women worldwide [1]. Due to the deficiency of effective methods for screening and atypical clinical symptoms in the early stage, the majority of ovarian cancer cases are usually diagnosed in the advanced stages [2, 3], and their 5-year survival rate is less than 50% [4]. Therefore, establishing primary prevention for ovarian cancer, although challenging, is a crucial part of public health.

Over the past decades, much evidence has indicated that adult obesity is an essential factor in the development of ovarian cancer [5–9], whereas little is known about the association between obesity in childhood and adolescence and ovarian cancer. Given the long latency of cancer development, the accumulation of risk factors over time from childhood and adolescence may be more closely associated with ovarian cancer than in adulthood [10]. From 1975 to 2016, the global prevalence of overweight or obesity among female children and adolescents aged 5‒19 years increased by eight times [11] and showed a continuously increasing trend [12]. A meta-analysis showed that obese children and adolescents were approximately five times more likely to be obese in adulthood than those who were not obese [13], which suggests that childhood and adolescence may be a critical window of ovarian carcinogenic susceptibility to obesity [14, 15]. However, the current findings of the association between obesity in childhood and adolescence and ovarian cancer are mixed. Some observational studies have shown that obesity in adolescence has a stronger association with ovarian cancer than that in adulthood [16, 17]. Other studies have reported the opposite that there is no significant association between obesity and ovarian cancer at age 18 [18, 19]. Thus, it is essential to verify the association between obesity in children and adolescents and ovarian cancer.

To provide more convincing epidemiological evidence for further studies, we performed this meta-analysis to better evaluate the association between obesity in children and adolescents and ovarian cancer and to analyze whether there is a dose‒response. Furthermore, a considerable number of predesignated subgroup analyses were carried out to identify covariates that may influence the pooled effect size estimates and test the stability of the results.

## Methods

### Guidelines and ethics

The reports in the study conformed to the guidelines in the Preferred Reporting Items for Systematic Reviews and Meta-analyses (PRISMA) statement [20]. The PRISMA checklist is presented in S1 Table. The study protocol was registered with the International Prospective Register of Systematic Reviews (PROSPERO), and the registration number is CRD42022331501. Since the present meta-analysis was based on published research, the approval and informed consent steps of the Ethics Committee were omitted.

### Search strategy

The literature search was conducted in the PubMed, Embase, Web of Science and Cochrane Library databases as of April 6, 2022, and was updated on October 5, 2022. The full search strategy is presented in the S2 Table. In addition, we manually searched the reference lists of the main references to avoid potential omissions. The included articles were not subject to language and publication restrictions. If necessary, we contacted the author to try to obtain more relevant information.

The search process was independently performed by two investigators (N.D. and J.Z.) using the same medical subject terms. All retrieved references were combined and duplicates were removed.

### Eligibility criteria

This meta-analysis included articles that met the following criteria: (ⅰ) Study participants: aged less than 19 years; (ⅱ) Endpoints: all types of ovarian cancer; (ⅲ) Study design: cohort studies or case‒control studies; (ⅳ) Exposure: overweight or obesity in children or adolescents; (ⅴ) Relative risk (RR), hazard ratio (HR) or odds ratio (OR) with 95% confidence interval (95% CI) was used to measure the degree of relationship between child and adolescent obesity and ovarian cancer.

Articles were excluded if they were case reports, case series, editorials, or narrative reviews, or if the participants belonged to a population with a specific disease. Articles where the full-text was not available or those not written in the English language were also excluded. Furthermore, when multiple studies were from the same cohort, we included only those with the longest follow-up period and the largest sample size.

### Data abstraction and quality assessment

We extracted the following data from each eligible study: (ⅰ) Characteristic information: first author, time of publication, country or region, study design, study period, follow-up year, baseline age, outcome, sample size, and number of cases. (ⅱ) Exposure evaluation: BMI or weight, or other measures used to assess obesity. Moreover, the reference categories of the definition or classification were treated as the benchmark for extracting data. (ⅲ) Effect size estimate: adjusted RRs, HRs or ORs with 95% CIs by categories.

In addition, we recorded the adjustment of covariates in each study in detail for subgroup analysis, such as age, duration of oral contraceptive use, age at menarche, menopausal status, family history, race, parity, and tubal ligation history.

Data were extracted independently from each included study by two investigators (N.D. and J.Z.). The divergence was resolved by the co-evaluation of original articles or by a third author if necessary (Y.S.).

The quality of all included studies was assessed by the Newcastle‒Ottawa Scale (NOS) for cohort and case‒control studies [21]. The evaluation of a study using the NOS is based on the following aspects: the selection and comparability of study groups, and the assessment of exposures and outcomes.

### Statistical analyses

Data management and analysis were analyzed by STATA 14.1. The effect size was estimated as RR, HR or OR with a 95% CI. We converted HRs into RRs and used ORs as approximations of RRs to combine effect values [22, 23]. The meta-regression analysis model was selected according to the results of the between-test heterogeneity. When the heterogeneity was significant, a random effect model was selected, and a fixed effect model was derived when the heterogeneity was not significant [24]. The Cochrane Q test and inconsistency index (*I*^2^) were used to quantify the heterogeneity between studies. For the Cochrane Q test, p<0.1 was considered statistically significant, and for the *I*^2^ statistic, *I*^2^ values greater than 50% were considered to indicate significant heterogeneity. Since there was no significant heterogeneity between studies, we derived pooled effect size estimates under the fixed model.

The generalized least squares regression proposed by Greenland and Longnecker was used to establish the dose‒response relationship. The median of each category of BMI was regarded as the exposure dose, and the length of the adjacent interval was treated as the length of the open interval. The restricted cubic splines of exposure distributions with three knots fixed at the 25^th^, 50^th^, and 75^th^ percentiles were used to test nonlinearity between obesity in children and adolescents and ovarian cancer.

To identify the factors that may affect the pooled effect size estimates and test the stability of the results, a considerable number of predesignated subgroup analyses were performed based on demographic characteristics, research characteristics, and whether the covariates were adjusted. A sensitivity analysis was used to assess the impact of each article on the overall stability.

The risk of publication bias was evaluated by Begg’s funnel plots and Egger regression asymmetry tests. The number of theoretically missing studies was estimated by the trim-and-fill method.

## Results

### Eligible studies

**Fig 1:**
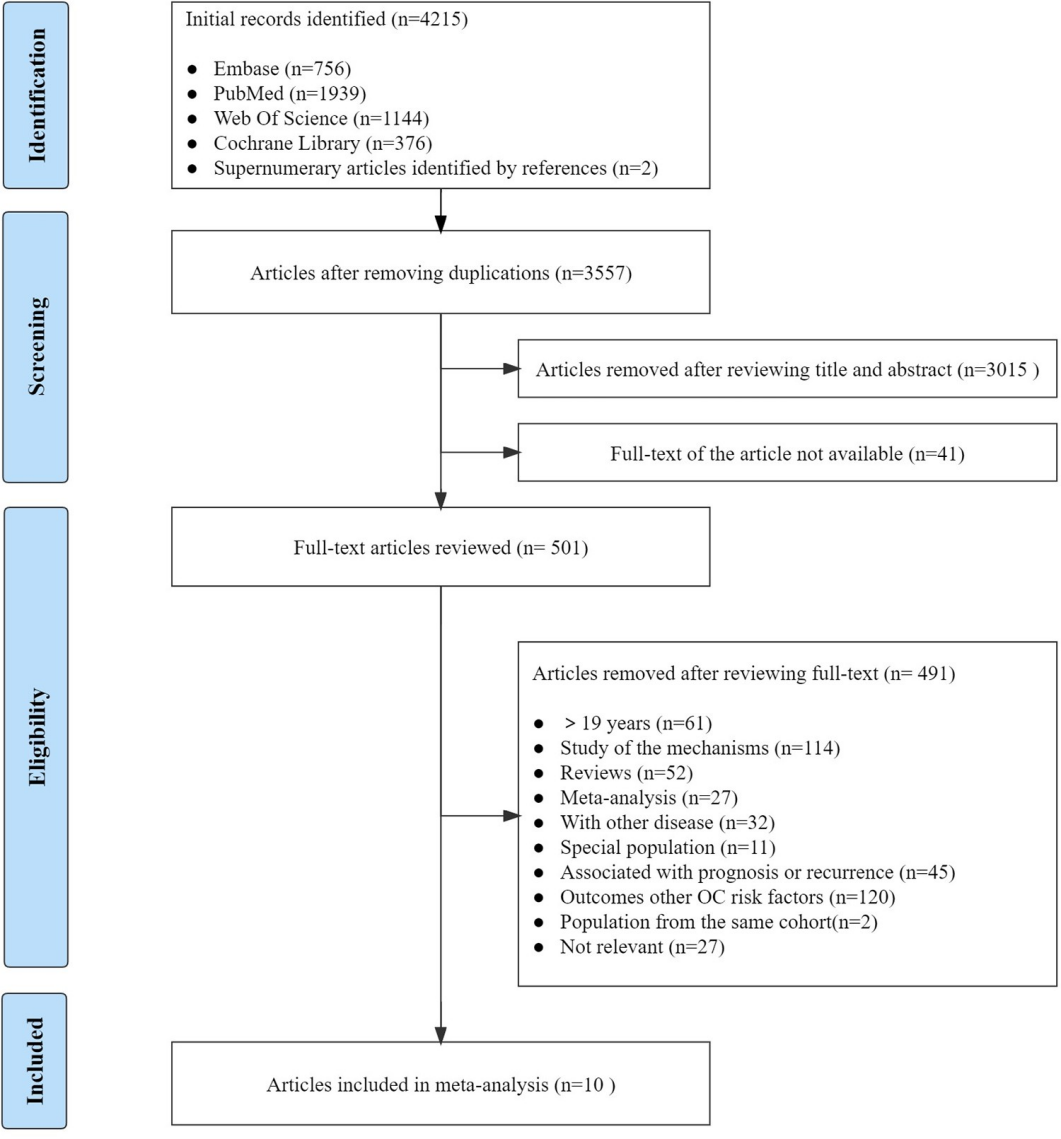
Flow chart of study selection in this meta-analysis.

A total of 4215 publications were initially identified by searching the medical subject words in the previously mentioned public databases, and 10 articles were selected [14, 16, 17, 25–31], including 588,134 participants. The results of some articles were reported by different age groups and these were listed and treated separately. The detailed search strategy, inclusion process, and exclusion process are presented in Fig 1.

### Study characteristics

The baseline characteristics of the 10 eligible articles including 5 cohort studies [14, 16, 17, 25, 28] and 5 case‒control studies [26, 27, 29–31], are presented in Table 1. The original data used to reach the conclusions were recorded on S3 Table to improve the repeatability of the present study. Depending on the measurements of body size, all studies reported BMI in children and adolescents, and 4 studies reported weight [26, 29–31]. According to age, 8 articles reported BMI at 18 years of age [16, 17, 26–31], 1 article reported BMI at 17 years of age [25], and 2 articles reported BMI at 10 years of age [14, 16]. Based on geographical location, 7 articles were from the Americas[16, 17, 26, 28–31], 2 articles were from Europe[14, 25] and 1 article was from Asia [27]. Meanwhile, we made a detailed classification of adjusted multiple covariates.

**Table 1:** Descriptive characteristics of studies on the association of obesity in children and adolescents with ovarian cancer.

First author	Year	Country	Study Design	Study period	Follow-up (years)	Exposure assessment	Age exposure assessed	Outcome	Adjusted variables
**Kuper, H.**	2002	US	C-C	1992–1997	NA	BMI, weight	18	Epithelial ovarian cancer	age, site, parity, oral contraceptive use, family history of breast, ovarian, or prostate cancer in a first-degree relative, tubal ligation, education, and marital status
**Lubin, F.**	2003	Israel	C-C	1994–1999	NA	BMI	18	Epithelial ovarian cancer	family history of either breast or ovarian cancer in first-degree family members, use of oral contraceptive, parity, menopausal status, age at menarche, infertility problems requiring treatment, change in body mass index
**Engeland, A.**	2003	Norway	Cohort	1963–1999	31.6	BMI	14–16, 17–19	ovarian cancer	age at measurement and birth cohort
**Anderson, J. P.**	2004	US	Cohort	1986–2000	15	BMI	18	Epithelial ovarian cancer	age, family history of ovarian cancer, hysterectomy status, oophorectomy status, number of live births, pack-years of smoking, and estrogen replacement therapy (ever/ never)
**Hoyo, C.**	2005	US	C-C	1999–2003	NA	BMI, weight	18	Epithelial ovarian cancer	race, age, parity, ovarian cancer history, breast cancer history, hysterectomy, oral contraceptive use, and menstrual status
**Greer, J. B.**	2006	US	C-C	1994–1998	NA	BMI, weight	18	Epithelial ovarian cancer	age, race (white/other), number of live births, family history of ovarian cancer, tubal ligation, and oral contraceptive use (ever/never)
**Rossing, M. A.**	2006	US	C-C	1994–1998	NA	BMI, weight	18	Epithelial ovarian cancer	age, race, study site, number full-term births and duration of oral contraceptive use
**Leitzmann, M. F.**	2009	US	Cohort	1996–2003	7	BMI	18	Epithelial ovarian cancer	age, race/ethnicity, family history of breast or ovarian cancer, duration of oral contraceptive use, menopausal hormone therapy, and physical activity
**Aarestrup, J.**	2019	Denmark	Cohort	1978–2014	37	BMI	7, 8, 9, 10, 11, 12, 13	Epithelial ovarian cancer	birth weight
**Huang, T.**	2019	US	Cohort	1980–2012 1989–2013	32, 24	BMI	10, 18	ovarian cancer	age at menarche, tubal ligation, duration of oral contraceptive use, duration of premenopause, menopausal status, type of menopause, duration of estrogen-only hormone therapy use, duration of estrogen and progesterone hormone therapy use, duration of other hormone therapy use, height, parity and smoking status

C-C: case–control study; BMI: body mass index

### Quality assessment

S4 and S5 Tables present the quality assessment of all eligible articles by using the NOS for cohort studies and case‒control studies. The average scores were 8 (range: 7 to 9) and 7 (range: 6 to 8) respectively, with a standard deviation of 0.71.

### Meta-analyses

**Fig 2:**
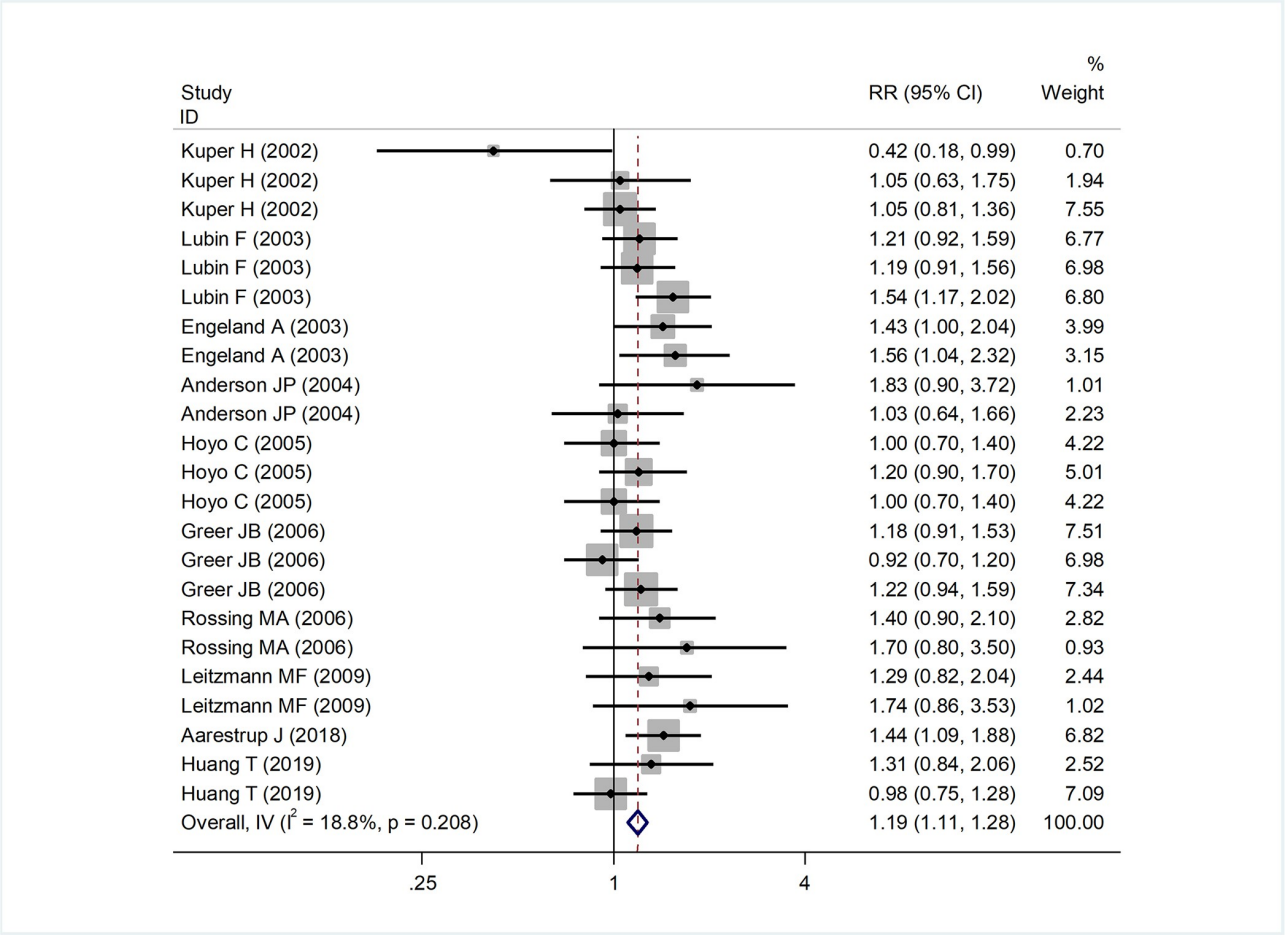
Association of obesity in children and adolescents with ovarian cancer in studies providing relative risks (RRs) and 95% confidence intervals (CIs).

After summarizing the results of all qualified studies, we speculated that obesity in children and adolescents was statistically associated with an increased risk of ovarian cancer (RR = 1.19, 95% CI: 1.11 to 1.28, P < 0.001). The estimated results displayed in Fig 2 addressed the association of obesity in children and adolescents with ovarian cancer.

A series of subgroup analyses were conducted to reveal the potential factors that may affect the pooled effect size estimates and test the stability of the results. The detailed overall and subgroup analyses are shown in Table 2. By study design, the association between obesity in children and adolescents and ovarian cancer was statistically significant in both cohort studies (RR = 1.29, 95% CI: 1.13 to 1.47, P < 0.001) and case‒control studies (OR = 1.15, 95% CI: 1.06 to 1.25, P = 0.001). By baseline age, the association of obesity in children and adolescents and ovarian cancer was significant at 18 years of age (RR = 1.15; 95% CI: 1.07 to 1.24; P = 0.001), while there was no significant association at 10 years of age (RR = 1.07; 95% CI: 0.95 to 1.20; P = 0.254). However, the pooled result at 10 years of age should be considered with caution due to the limited number of included studies (n = 2). By exposure assessment, the significant association between obesity in children and adolescents and ovarian cancer was consistent both for researchers’ assessment (RR = 1.46; 95% CI: 1.21 to 1.77; P<0.001) and self-report (RR = 1.15; 95% CI: 1.07 to 1.24; P = 0.001). By geographic region, ovarian cancer risk was significantly associated with obesity in children and adolescents in all subgroups (America: RR = 1.11; 95% CI: 1.01 to 1.21; P = 0.022, Europe: RR = 1.46; 95% CI: 1.21 to 1.77; P<0.001). By body size evaluation methods, in addition to BMI, excessive weight also showed a significant correlation with ovarian cancer (OR = 1.14; 95% CI: 1.04 to 1.25; P = 0.004). To explore important covariates that may affect the association between obesity in children and adolescents and ovarian cancer risk, we further pooled the estimated effect sizes of all studies that adjusted the same covariable. The results indicate that the association remained significantly positive in most subgroups after adjusting for covariables, including age, race, duration of oral contraceptive use, age at menarche, menopausal status, family history, and parity. These results suggested that obesity in children and adolescents was an independent risk factor for ovarian cancer.

**Table 2:** Overall and subgroup analyses of the association of obesity in children and adolescents with ovarian cancer risk.

Groups	Number of qualified studies	Effect size	95% CI	*p*	*I* ^2^
**Overall analyses**					
Risk of ovarian cancer	10	1.19	1.11–1.28	<0.001	18.80%
**Subgroup analyses**					
**Study design**					
Cohort	5	1.29	1.13–1.47	<0.001	4.30%
C-C	5	1.15	1.06–1.25	0.001	21.90%
**Measurement of body size**					
BMI	10	1.19	1.11–1.28	<0.001	18.80%
Weight	4	1.14	1.04–1.25	0.004	0.00%
**Baseline age**					
18	8	1.15	1.07–1.24	0.001	12.70%
17	1	1.49	1.14–1.94	-	-
10	2	1.07	0.95–1.20	0.254	19.40%
**Geographic region**					
America	7	1.11	1.01–1.21	0.022	2.90%
Europe	2	1.46	1.21–1.77	<0.001	0.00%
Asia	1	1.30	1.11–1.53	-	-
**Exposure assessment**					
Self-reported	8	1.15	1.07–1.24	0.001	12.70%
By researchers	2	1.46	1.21–1.77	<0.001	0.00%
**Adjusted for covariates**					
age	8	1.19	1.10–1.29	<0.001	17.40%
Duration of oral contraceptive use	7	1.15	1.06–1.24	0.001	14.60%
Age at menarche	2	1.22	1.07–1.39	0.003	27.40%
Menopausal status	3	1.17	1.05–1.30	0.004	6.60%
Family history	5	1.17	1.07–1.28	0.001	28.20%
Parity	7	1.14	1.06–1.24	0.001	15.70%
Tubal ligation history	3	1.06	0.95–1.19	0.283	18.00%
Smoking	2	1.11	0.90–1.38	0.334	10.50%
Race	3	1.12	1.00–1.26	0.048	0.00%

C-C: case–control study; BMI: body mass index.

A sensitivity analysis was conducted by omitting each article sequentially to further confirm the stability of the results, which revealed that none of these studies had a significant impact on the pooled estimate effect size (S1 Fig).

### Dose‒response analyses

**Fig 3:**
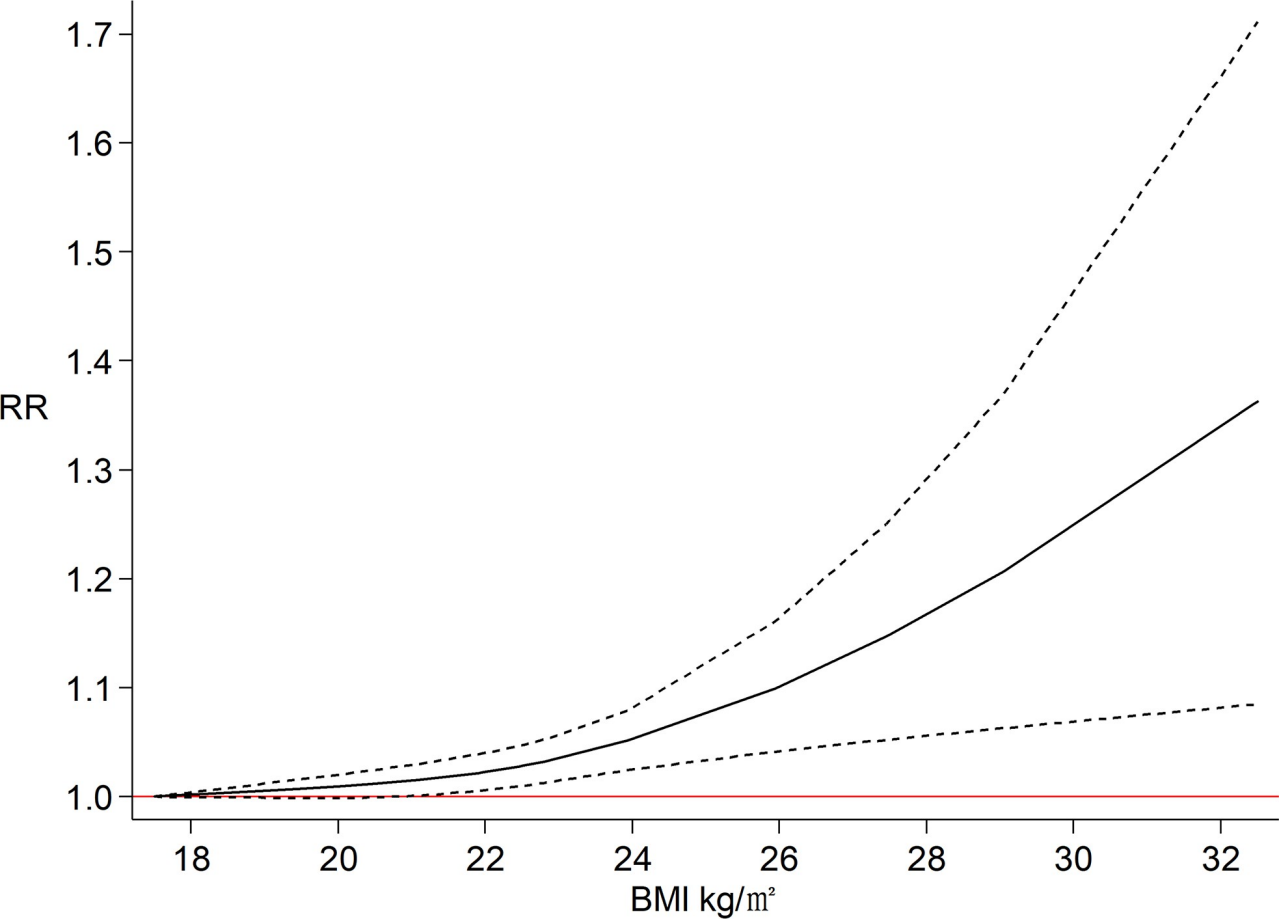
The dose‒response plot for the association of obesity in children and adolescents with ovarian cancer.

The estimated effect sizes of ovarian cancer risk by BMI or weight category are presented in S6 Table, indicating that both BMI and weight show a higher risk of ovarian cancer with a higher level of adiposity. The dose‒response relationship between BMI in children and adolescents and ovarian cancer is illustrated in Fig 3 and shows a J-distribution, indicating that the ovarian cancer risk would increase rapidly when BMI is greater than 25.95 kg/m^2^.

### Publication bias

Begg’s and filled funnel plots were used to assess publication bias for the association of obesity in children and adolescents with ovarian cancer and are presented in S2 Fig. There was no asymmetry evidence of study effects in Begg’s test and further Egger’s tests confirmed that (P = 0.650). The filled funnel plots revealed 3 missing items in the theory study.

## Discussion

To the best of our knowledge, this meta-analysis is the first assessment exploring the association between obesity in children and adolescents and ovarian cancer. Ten eligible studies with more than 500,000 participants were included in this meta-analysis. Our key findings are that children and adolescents with obesity had a 1.19 times higher risk of ovarian cancer than those without obesity. When considering the adjustment of covariables, the association was consistently significant in most subgroups, suggesting that the associations were independent of these risk factors. Moreover, the J-distribution dose‒response in the relationship between BMI in children and adolescents and ovarian cancer indicates that ovarian cancer risk increases with more serious obesity in children and adolescents. Our findings suggest that it is necessary to pay more attention to the control of obesity in children and adolescents to prevent ovarian cancer.

In previous literature, some prospective cohort studies had similar conclusions that obesity in children and adolescents could be a risk factor for ovarian cancer [14, 25]. However, several observational studies have shown controversial findings. A case‒control study including 1988 participants in the United States found that there was no statistical association between obesity at the age of 18 as measured by BMI and ovarian cancer (adjusted OR = 1.7; 95% CI: 0.8 to 3.5). However, this association was significant relative to low weight compared with high weight at the age of 18 (adjusted OR = 1.5; 95% CI: 1.0 to 2.2) [31]. Similarly, Fairfield advocated that there was no statistically significant association between overweight at the age of 18 and ovarian cancer (adjusted RR = 1.12; 95% CI: 0.77 to 1.63) through the analysis of 109,445 participants in the Nurses’ Health Study [18]. Recently, a meta-analysis from Byun and colleagues suggested that the risk of ovarian cancer increased each 15% when early-life BMI increases every 5 kg/m^2^, (RR = 1.15; 95% CI: 1.07 to 1.23), which provided high-level evidence that early-life obesity could be a significant risk factor for ovarian cancer [32]. Nevertheless, the number of included studies (6 cohort studies) was insufficient, and the data were mixed with the early adulthood results (age ≤ 25 years). Therefore, the association between obesity in children and adolescents and ovarian cancer could not be accurately interpreted.

To fill the research gap, the age of participants was restricted to less than 19 years old to focus on the association between obesity in children and adolescents and ovarian cancer. For the selection of literature, cohort studies and case‒control studies were included to expand the sample size and diminish random error and selection bias. For the combination of estimated effect sizes, using multifactor adjusted effect values may better reduce the influence of covariates on the results. For the subgroup analyses, detailed classification was performed to expose potential factors that might affect the conclusions and test the stability of the results. Furthermore, the dose‒response analysis described the association between BMI in children and adolescents and ovarian cancer risk.

Our study provided evidence to support the long-term effects of obesity in children and adolescents associated with the risk of ovarian cancer. The pooled estimate effect sizes of both cohort studies and case‒control studies showed statistically significant associations. The dose‒response findings further confirmed our conclusion, that ovarian cancer risk increases with increasing BMI in children and adolescents, and ovarian cancer risk rapidly increases when BMI exceeds 25.95 kg/m^2^. It is worth noting that, participants in late adolescence at the ages of 17 (RR = 1.49; 95% CI: 1.14 to 1.94) and 18 (RR = 1.15; 95% CI: 1.07 to 1.24) showed significant associations between obesity and ovarian cancer when stratified by age. However, there was no association at the age of 10 (RR = 1.07; 95% CI: 0.95 to 1.20). BMI at the age 10 was the result of body size estimation, which may cause information bias.

Until now, the biological mechanisms of increased ovarian cancer risk with obesity in children and adolescents have not been fully elucidated. Weight and BMI gain among children and adolescents may reflect prolonged exposure to obesity and continuous accumulation of risk factors throughout the life course, thus making children and adolescents more prone to the carcinogenic process. Obesity is a pathological state accompanied by overgrowth of adipose tissue. Adipocytes, the major component of adipose tissue, are involved in almost all ovarian cancer processes by secreting adipokines, metabolic remodeling and regulating the immune microenvironment, which can promote the growth, invasion, metastasis and angiogenesis of ovarian cancer [33]. On the one hand, adipocytes provide ovarian cancer with high-energy metabolites through altered lipid metabolism and resistance to mitochondrial apoptosis, which is one of the primordial drivers of cancer cell growth and proliferation [34, 35]. On the other hand, the immune microenvironment is considered a major factor in the development and progression of ovarian cancer. During weight gain, adipocytes contribute to the release of inflammatory factors such as tumor necrosis factor-α, interleukin (IL)-6 and IL-1β from adipose tissue macrophages via paracrine pathways, inducing a chronic low-grade inflammatory microenvironment that stimulates persistent abnormal proliferation of ovarian epithelial cells [36, 37]. In addition, adipocytes can inhibit the antitumor activity of immune cells by expressing programmed death-ligand 1, allowing cancer cells to evade immune surveillance [38]. In obese populations, the excessive accumulation of adipocytes exerts a chronic and persistent effect on the immune microenvironment, which may lead to a higher risk of cancer in children and adolescents exposed to long-term persistent obesity than in adults. Meanwhile, obesity is associated with higher levels of androgens [39], and ovarian cancer preferentially develops in an androgen-rich hormonal environment [40]. Excess androgens may directly affect ovarian cancer development through androgen receptor signaling [41]. Puberty is accompanied by increased androgen secretion, and total testosterone levels are significantly higher in obese girls during puberty than in normal weight girls, which may further increase the risk of ovarian cancer [42, 43]. This biological change is consistent with our conclusion that there is a statistically significant association between obesity in puberty and ovarian cancer risk. From another point of view, the incessant ovulation hypothesis suggests that repeated ovarian epithelial damage and repair due to constant ovulation is an important risk factor for ovarian cancer, and therefore women with a high lifetime number of ovulatory cycles are at higher risk of ovarian cancer [44, 45]. Excessive body fat may lead to earlier puberty in girls [46] and contribute to increasing the lifetime number of ovulatory cycles, which may also be critical to the risk of ovarian cancer [15, 47]. Under the background of long-term follow-up, anthropometric data for children and adolescents are difficult to obtain, which hinders research on relevant biological mechanisms. In the future, further large and well‐designed observational studies are needed to validate current theories.

There are also some limitations in this study that need to be addressed. First, our findings suggest that obesity in children and adolescents increases the risk of ovarian cancer. However, due to the lack of relevant literature containing childhood obesity data, the analysis of dose‒response relationships by multiple age groups could not be performed. Therefore, more measurements across different age groups are needed to prove our results. Second, subgroup analyses indicated that most studies were performed in the Americas, which may be related to higher obesity rates in children and adolescents in the Americas [48], implying that the universality and difference of this association cannot be reasonably explained across multiple ethnic backgrounds. Third, there was no correction applied for adult obesity in this meta-analysis, because the original data from the included articles did not adjust for this factor.

## Conclusion

Our dose‒response meta-analysis supports that obesity in children and adolescents may independently increase the risk of ovarian cancer and that the degree of risk increases with the severity of obesity. However, the relationship with obesity in childhood is not clear. Future research through more large-scale, well-designed cohort studies on the relationship between childhood obesity and ovarian cancer is needed.

## Supporting information

S1 Table PRISMA checklist for systematic review. (DOCX)

S2 Table Search strategies for databases. (DOCX)

S3 Table Original data extracted from included studies. (DOCX)

S4 Table Newcastle‒Ottawa scale for assessment of quality of cohort studies. (DOCX)

S5 Table Newcastle‒Ottawa scale for assessment of quality of case–control studies. (DOCX)

S6 Table Estimate effect sizes of ovarian cancer risks by BMI or weight category. (DOCX)

S1 Fig Sensitivity analysis. (DOCX)

S2 Fig Begg’s and filled funnel plots for the association of obesity in children and adolescents with ovarian cancer. (DOCX)
