# Supplementary material for: Obesity in children and adolescents and the risk of ovarian cancer: A systematic review and dose‒response meta-analysis
Source: PLoS One. 2022 Dec 7;17(12):e0278050. doi: 10.1371/journal.pone.0278050 (PMC9728843; doi:10.1371/journal.pone.0278050)
Supplement: S2 Table — (DOCX) [file pone.0278050.s002.docx]

**S2 Table. Search strategies for databases.**

| **Pubmed**  **(n=1939)** | (adolescent* OR teen* OR youth* OR child* OR early life OR pediatric OR early adulthood) AND (ovarian neoplasm* OR ovary neoplasm* OR ovary cancer* OR ovarian cancer* OR cancer of ovary OR ovarian malignancy OR ovarian tumor) AND (obesity OR adiposity OR overweight OR body fat distribution OR body fat patterning OR body shape* OR somatotype OR body type* OR body build* OR body fatness OR body mass index OR weight OR body size* OR BMI) [All Fields] |
| --- | --- |
| **EMBASE**  **（n=756）** | (adolescent* OR teen* OR youth* OR child* OR 'early life'/exp OR 'pediatric'/exp OR 'early adulthood') AND ('ovarian neoplasm*' OR 'ovary neoplasm*' OR 'ovary cancer*' OR 'ovarian cancer*' OR 'cancer of ovary' OR 'ovarian malignancy'/exp OR 'ovarian tumor'/exp) AND ('obesity'/exp OR 'adiposity'/exp OR 'overweight'/exp OR 'body fat distribution'/exp OR 'body fat patterning' OR 'body shape*' OR 'somatotype'/exp OR 'body type*' OR 'body build*' OR 'body fatness' OR 'body mass index'/exp OR 'weight'/exp OR 'body size*' OR 'bmi'/exp) |
| **Web of Science**  **（n=1144）** | ((ALL=(adolescent* OR teen* OR youth* OR child* OR early life OR pediatric OR early adulthood)) AND ALL=(ovarian neoplasm* OR ovary neoplasm* OR ovary cancer* OR ovarian cancer* OR cancer of ovary OR ovarian malignancy OR ovarian tumor)) AND ALL=(obesity OR adiposity OR overweight OR body fat distribution OR body fat patterning OR body shape* OR somatotype OR body type* OR body build* OR body fatness OR body mass index OR weight OR body size* OR BMI) |
| **Cochrane Library**  **（n=376）** | (adolescent* OR teen* OR youth* OR child* OR early life OR pediatric OR early adulthood) AND (ovarian neoplasm* OR ovary neoplasm* OR ovary cancer* OR ovarian cancer* OR cancer of ovary OR ovarian malignancy OR ovarian tumor) AND (obesity OR adiposity OR overweight OR body fat distribution OR body fat patterning OR body shape* OR somatotype OR body type* OR body build* OR body fatness OR body mass index OR weight OR body size* OR BMI) |
