# Supplementary material for: Obesity in children and adolescents and the risk of ovarian cancer: A systematic review and dose‒response meta-analysis
Source: PLoS One. 2022 Dec 7;17(12):e0278050. doi: 10.1371/journal.pone.0278050 (PMC9728843; doi:10.1371/journal.pone.0278050)
Supplement: S3 Table — (DOCX) [file pone.0278050.s003.docx]

**S3 Table (a). Original data extracted from included studies (Using BMI measurement).**

| **First author** | **Year** | **Baseline age** | **Effect estimate** | **Sample size** | **Cases** | **categories** | **Effect size** | **Low limit** | **High limit** |
| --- | --- | --- | --- | --- | --- | --- | --- | --- | --- |
| Kuper, H. | 2002 | 18 | OR | 1089 | 563 | 20-25 | 1.05 | 0.81 | 1.36 |
| Kuper, H. | 2002 | 18 | OR | 1089 | 563 | 25-30 | 1.05 | 0.63 | 1.75 |
| Kuper, H. | 2002 | 18 | OR | 1089 | 563 | ≥30 | 0.42 | 0.18 | 0.99 |
| Lubin, F. | 2003 | 18 | OR | 3380 | 1269 | 19.1-20.9 | 1.19 | 0.91 | 1.56 |
| Lubin, F. | 2003 | 18 | OR | 3380 | 1269 | 21.0-22.8 | 1.21 | 0.92 | 1.59 |
| Lubin, F. | 2003 | 18 | OR | 3380 | 1269 | 22.9-35.2 | 1.54 | 1.17 | 2.02 |
| Engeland, A. | 2003 | 17 | RR | 111883 | 260 | 75th-84th | 1.43 | 1 | 2.04 |
| Engeland, A. | 2003 | 17 | RR | 111883 | 260 | ≥85th | 1.56 | 1.04 | 2.32 |
| Anderson, J. P. | 2004 | 18 | RR | 41836 | 222 | 25-30 | 1.03 | 0.64 | 1.66 |
| Anderson, J. P. | 2004 | 18 | RR | 41836 | 222 | ≥30 | 1.83 | 0.90 | 3.72 |
| Hoyo, C. | 2005 | 18 | OR | 1221 | 575 | 18.531–20.010 | 1 | 0.7 | 1.4 |
| Hoyo, C. | 2005 | 18 | OR | 1221 | 575 | 20.011–21.679 | 1 | 0.7 | 1.4 |
| Hoyo, C. | 2005 | 18 | OR | 1221 | 575 | >21.679 | 1.2 | 0.9 | 1.7 |
| Greer, J. B. | 2006 | 18 | OR | 2110 | 759 | 18.7–20.2 | 0.92 | 0.7 | 1.2 |
| Greer, J. B. | 2006 | 18 | OR | 2110 | 759 | 20.3–21.9 | 1.22 | 0.94 | 1.59 |
| Greer, J. B. | 2006 | 18 | OR | 2110 | 759 | ＞22.0 | 1.18 | 0.91 | 1.53 |
| Rossing, M. A. | 2006 | 18 | OR | 1988 | 353 | 25-30 | 1.4 | 0.9 | 2.1 |
| Rossing, M. A. | 2006 | 18 | OR | 1988 | 353 | ≥30 | 1.7 | 0.8 | 3.5 |
| Leitzmann, M. F. | 2009 | 18 | RR | 84211 | 271 | 25.0-29.9 | 1.29 | 0.82 | 2.04 |
| Leitzmann, M. F. | 2009 | 18 | RR | 84211 | 271 | ≥30 | 1.74 | 0.86 | 3.53 |
| Aarestrup, J. | 2019 | 13 | HR | 102287 | 630 | Overweight | 1.44 | 1.09 | 1.89 |
| Huang, T. | 2019 | 18 | HR | 238129 | 788 | 25-29.9 | 0.98 | 0.75 | 1.29 |
| Huang, T. | 2019 | 18 | HR | 238129 | 788 | ≥30 | 1.32 | 0.84 | 2.08 |

RR, relative risk; OR: odds ratio; HR: hazard ratio.

**S3 Table (b). Original data extracted from included studies (Using weight measurement).**

| **First author** | **Year** | **Baseline age** | **Effect estimate** | **Sample size** | **Cases** | **categories** | **Effect size** | **Low limit** | **High limit** |
| --- | --- | --- | --- | --- | --- | --- | --- | --- | --- |
| Kuper, H. | 2002 | 18 | OR | 1089 | 563 | 49.9-54.3 | 0.88 | 0.61 | 1.27 |
| Kuper, H. | 2002 | 18 | OR | 1089 | 563 | 54.4-59.0 | 1.04 | 0.71 | 1.52 |
| Kuper, H. | 2002 | 18 | OR | 1089 | 563 | ≥59.0 | 1 | 0.7 | 1.44 |
| Hoyo, C. | 2005 | 18 | OR | 1221 | 575 | 48-52 | 1 | 0.7 | 1.4 |
| Hoyo, C. | 2005 | 18 | OR | 1221 | 575 | 53-57 | 1 | 0.7 | 1.4 |
| Hoyo, C. | 2005 | 18 | OR | 1221 | 575 | ＞57 | 1.4 | 1 | 1.9 |
| Greer, J. B. | 2006 | 18 | OR | 2110 | 759 | 49.5-54.0 | 1.07 | 0.83 | 1.37 |
| Greer, J. B. | 2006 | 18 | OR | 2110 | 759 | 54.1-58.5 | 1.32 | 1.01 | 1.74 |
| Greer, J. B. | 2006 | 18 | OR | 2110 | 759 | ＞58.5 | 1.38 | 1.06 | 1.80 |
| Rossing, M. A. | 2006 | 18 | OR | 1988 | 353 | 44.90-54.43 | 1 | 0.7 | 1.4 |
| Rossing, M. A. | 2006 | 18 | OR | 1988 | 353 | 54.88-59.87 | 1 | 0.7 | 1.5 |
| Rossing, M. A. | 2006 | 18 | OR | 1988 | 353 | 60.33-67.59 | 1.3 | 0.8 | 1.9 |
| Rossing, M. A. | 2006 | 18 | OR | 1988 | 353 | ＞68.04 | 1.5 | 1 | 2.2 |

OR: odds ratio.
