# Supplementary material for: Obesity in children and adolescents and the risk of ovarian cancer: A systematic review and dose‒response meta-analysis
Source: PLoS One. 2022 Dec 7;17(12):e0278050. doi: 10.1371/journal.pone.0278050 (PMC9728843; doi:10.1371/journal.pone.0278050)
Supplement: S4 Table — (DOCX) [file pone.0278050.s004.docx]

**S4 Table.** **Newcastle‒Ottawa Scale for Assessment of Quality of Cohort Studies.**

| **Study** | **Selection** | | | | **Comparability** | | **Outcome** | | | **Total score** |
| --- | --- | --- | --- | --- | --- | --- | --- | --- | --- | --- |
|  | Representativeness of the exposed cohort | Selection of the non- exposed cohort | Ascertainment of exposure | Demonstration that outcome of interest was not present at start of study | Control for important factors | Control for important additional factors | Assessment of outcome | Was follow-up long enough for outcomes to occur | Adequacy of follow up of cohorts |  |
| Engeland, A. 2003 | 1 | 1 | 1 | 1 | 1 | 1 | 1 | 1 | 1 | 9 |
| Anderson, J. P. 2004 | 1 | 1 | 0 | 1 | 1 | 1 | 1 | 1 | 1 | 8 |
| Leitzmann, M. F. 2009 | 1 | 1 | 0 | 1 | 1 | 1 | 1 | 1 | 1 | 8 |
| Aarestrup, J. 2019 | 1 | 1 | 1 | 1 | 1 | 0 | 1 | 1 | 1 | 8 |
| Huang, T. 2019 | 0 | 1 | 0 | 1 | 1 | 1 | 1 | 1 | 1 | 7 |
