# Supplementary material for: Obesity in children and adolescents and the risk of ovarian cancer: A systematic review and dose‒response meta-analysis
Source: PLoS One. 2022 Dec 7;17(12):e0278050. doi: 10.1371/journal.pone.0278050 (PMC9728843; doi:10.1371/journal.pone.0278050)
Supplement: S5 Table — (DOCX) [file pone.0278050.s005.docx]

**S5 Table.** **Newcastle‒Ottawa Scale for Assessment of Quality of Case–Control Studies.**

| **Study** | **Selection** | | | | **Comparability** | | **Exposure** | | | **Total score** |
| --- | --- | --- | --- | --- | --- | --- | --- | --- | --- | --- |
|  | Adequate definition of the cases | Representativeness of the cases | Selection of controls | Definition of controls | Control for important factors | Control for important additional factors | Assessment of exposure | Same method of ascertainment for cases and controls | Non-Response rate |  |
| Kuper, H. 2002 | 1 | 1 | 1 | 1 | 1 | 1 | 1 | 1 | 0 | 8 |
| Lubin, F. 2003 | 1 | 1 | 1 | 1 | 1 | 1 | 0 | 1 | 0 | 7 |
| Hoyo, C. 2005 | 1 | 1 | 1 | 1 | 1 | 1 | 0 | 1 | 0 | 7 |
| Greer, J. B. 2006 | 1 | 1 | 0 | 1 | 1 | 1 | 0 | 1 | 0 | 6 |
| Rossing, M. A. 2006 | 1 | 1 | 1 | 1 | 1 | 1 | 0 | 1 | 0 | 7 |
