# Supplementary material for: Obesity in children and adolescents and the risk of ovarian cancer: A systematic review and dose‒response meta-analysis
Source: PLoS One. 2022 Dec 7;17(12):e0278050. doi: 10.1371/journal.pone.0278050 (PMC9728843; doi:10.1371/journal.pone.0278050)
Supplement: S6 Table — (DOCX) [file pone.0278050.s006.docx]

**S6 Table.** **Estimate effect sizes of ovarian cancer risks by BMI or weight category**

| **Measurement of body size** | **Number of qualified studies** | **Effect estimate** | **Effect size** | **95% CI** | ***p*** | ***I*^2^** |
| --- | --- | --- | --- | --- | --- | --- |
| **BMI** | | | | | | |
| Highest quantile (versus lowest quantile) | 8 | RR | 1.36 | 1.11-1.68 | 0.003 | 37.60% |
| Mid quantile (versus lowest quantile) | 8 | RR | 1.13 | 1.03-1.24 | 0.013 | 0.00% |
| **Weight** | | | | | | |
| Highest quantile (versus lowest quantile) | 4 | OR | 1.32 | 1.12-1.55 | 0.001 | 0% |
| Mid quantile (versus lowest quantile) | 4 | OR | 1.07 | 0.96-1.20 | 0.226 | 0% |

RR, relative risk; OR: odds ratio; BMI: body mass index.
