# Supplementary figures and images for: Obesity in children and adolescents and the risk of ovarian cancer: A systematic review and dose‒response meta-analysis
Source: PLoS One. 2022 Dec 7;17(12):e0278050. doi: 10.1371/journal.pone.0278050 (PMC9728843; doi:10.1371/journal.pone.0278050)

**S1 Fig. Sensitivity analysis.**

**
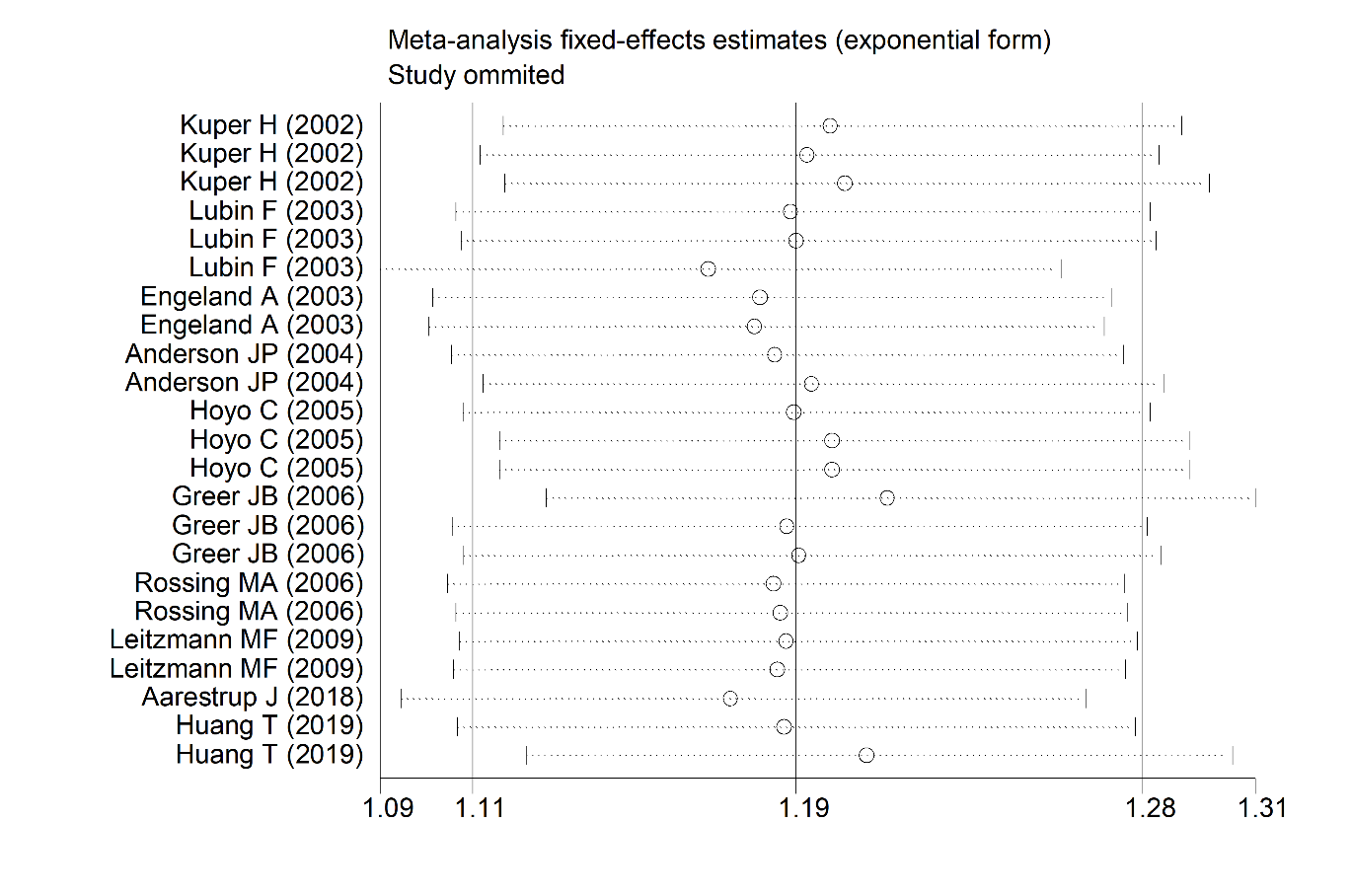
**

Supplement: S1 Fig — (DOCX) [file pone.0278050.s007.docx]
