# Supplementary material for: Obesity in children and adolescents and the risk of ovarian cancer: A systematic review and dose‒response meta-analysis
Source: PLoS One. 2022 Dec 7;17(12):e0278050. doi: 10.1371/journal.pone.0278050 (PMC9728843; doi:10.1371/journal.pone.0278050)
Supplement: S2 Fig — (DOCX) [file pone.0278050.s008.docx]

**S2 Fig. Begg's and filled funnel plots for the association of obesity in children and adolescents with ovarian cancer.**

**
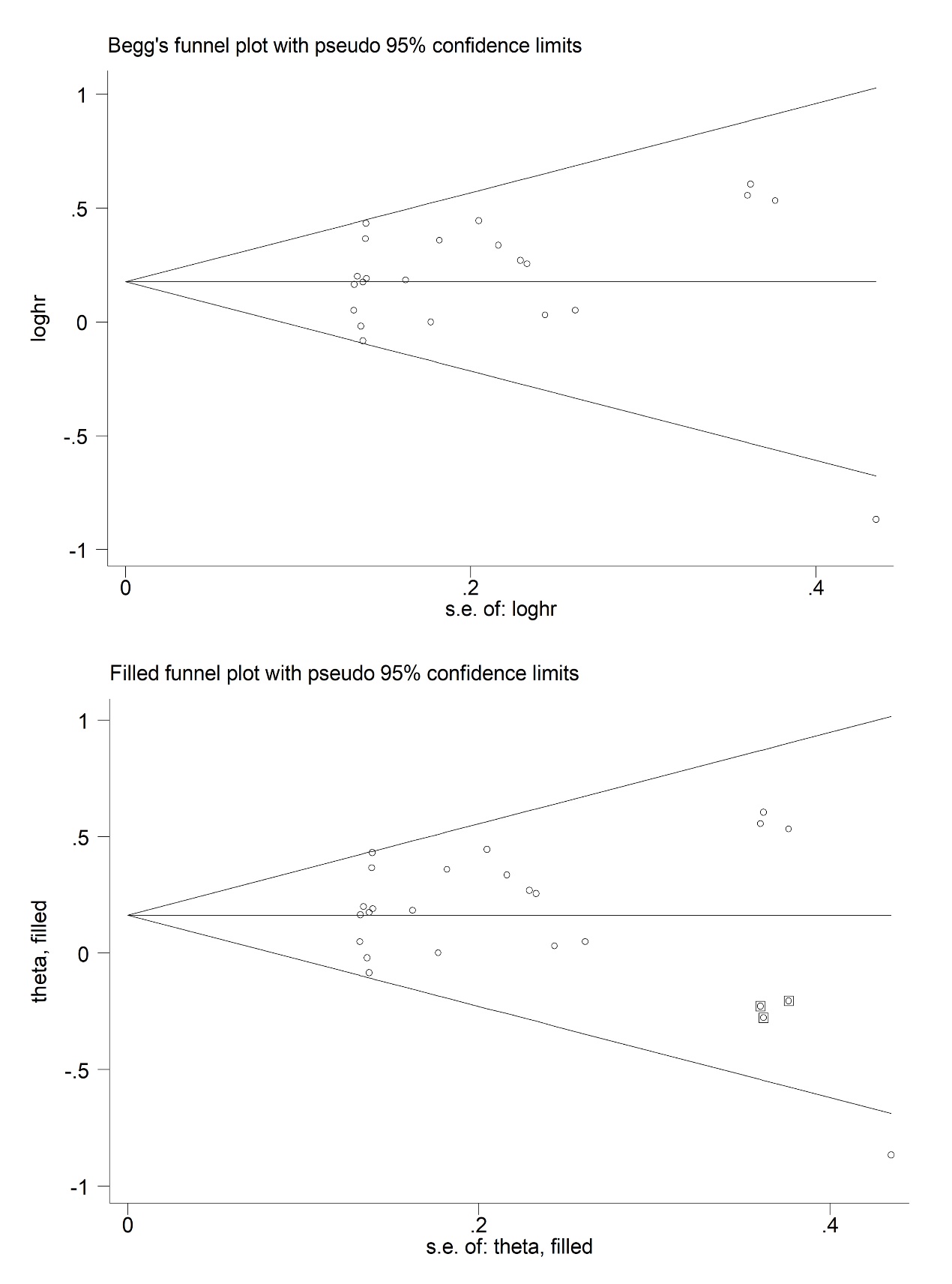
**
